# Dual‐Physical‐Field Nanocatalysis: Injectable Hydrogel Enables Piezo‐Photothermal Synergy for Breast Cancer Therapy

**DOI:** 10.1002/advs.202522447

**Published:** 2026-04-13

**Authors:** Can Tian, Shihan Xiao, Xuan Chen, Danni Zhang, Zizhen Liu, Jing Wang, Ning Xie, Wei Li

**Affiliations:** ^1^ Department of Breast Cancer Medical Oncology Hunan Cancer Hospital Changsha China; ^2^ The Affiliated Cancer Hospital of Xiangya School of Medicine Central South University / Hunan Cancer Hospital Changsha China; ^3^ Department of Breast and Thyroid Surgery The Second Affiliated Hospital University of South China Hengyang China; ^4^ Department of Oncology Hunan Institute of Schistosomiasis Control (The Third People's Hospital of Hunan Province) Yueyang China

**Keywords:** near‐infrared, piezocatalytic and photothermal therapy, reactive oxygen species, ultrasound, breast cancer

## Abstract

Piezocatalytic therapy (PCT) harnesses mechanical energy to generate tumor‐lethal reactive oxygen species (ROS), but its efficacy is limited by rapid electron‐hole recombination and poor intratumoral retention. To overcome these limitations, we engineered heterostructured BiOCl@CuO nanosheets embedded in an injectable, conductive, thermosensitive hydrogel composed of Pluronic F127 (F127) and reduced graphene oxide (rGO), with rGO specifically enhancing the hydrogel's mechanical strength and conductivity, for the combinational treatment of breast cancer. Under ultrasound (US) stimulation, BiOCl@CuO establishes a strong interfacial electric field that enhances charge separation and accelerates the generation of ROS. In addition, near‐infrared (NIR) irradiation activates BiOCl@CuO to convert light into heat, elevating intratumoral temperature and further amplifying ROS‐mediated cytotoxicity while enabling photothermal therapy (PTT). The integrated BiOCl@CuO/F127@rGO hydrogel exhibits rapid sol–gel transition at physiological temperature, robust tissue adhesion, and sustained local retention. In vitro, the synergistic therapy induced marked ROS bursts and achieved 60% breast cancer cell ablation at 50 µg/mL with less than 10% toxicity to normal cells. In vivo, orthotopic breast tumors treated with US+NIR showed 90% regression, reduced proliferation and angiogenesis, activation of apoptotic and immunogenic cell death pathways, and favorable biocompatibility. Critically, the regimen triggered robust local immune activation, increasing intratumoral CD8^+^ T cell infiltration by 2.7‐fold and IFN‐γ secretion by 5‐fold while upregulating DC maturation markers (CD80/CD86+) by 3‐fold. This work establishes a precise, minimally invasive strategy that couples piezocatalysis with photothermal conversion for effective breast cancer therapy.

## 1 Introduction

Breast cancer stands as the most prevalent malignancy among women worldwide, posing a severe threat to their health [1]. Although conventional therapies like surgery, chemotherapy, and radiotherapy have been extensively utilized, their therapeutic efficacy is often undermined by issues such as tumor recurrence and metastasis, treatment resistance, and inevitable systemic toxicity [2, 3, 4]. Recently, piezocatalytic therapy (PCT) has demonstrated considerable potential in tumor treatment as an emerging therapeutic modality driven by mechanical energy [5, 6]. PCT utilizes piezoelectric nanomaterials that drive electrons and holes apart under ultrasound (US) stimulation [7], reacting with water and oxygen to generate reactive oxygen species (ROS), such as hydroxyl radicals (·OH) and singlet oxygen (^1^O_2_) [8, 9]. These ROS inflict oxidative damage on cancer cells, causing cell death. However, PCT efficacy is limited by insufficient separation efficiency of electron‐hole pairs and rapid recombination of carriers upon US excitation.

Heterojunction engineering has emerged as a powerful strategy to improve piezocatalytic performance [10, 11, 12]. The built‐in electric field at heterojunction interfaces promotes efficient separation of US‐triggered charge carriers, thereby enhancing ROS generation [13]. This can be synergistically coupled with photothermal therapy (PTT), where near‐infrared (NIR) irradiation activates nanomaterials to induce localized hyperthermia [14, 15]. PTT leverages NIR irradiation to activate photothermal nanomaterials, inducing localized hyperthermia that directly ablates cancer cells [16]. The combination enables simultaneous oxidative stress and thermal damage, with hyperthermia potentiating ROS effects while ROS compensates for thermal limitations and can trigger anti‐tumor immune responses [17, 18].

Despite these advantages, existing piezocatalytic‐photothermal systems face critical limitations that hinder clinical translation. BaTiO_3_‐based materials exhibit strong piezoelectricity but suffer from poor NIR absorption and limited biodegradability [19, 20]. Metal‐organic framework (MOF)‐based systems offer high surface area but demonstrate poor physiological stability and concerns over metal ion leaching [21, 22]. Bi_2_WO_6_‐based heterostructures show good photocatalytic activity but weak piezoelectric response and require Ultraviolet (UV) excitation, which has poor tissue penetration [23]. Moreover, most reported systems utilize free nanoparticles that are rapidly cleared by the reticuloendothelial system, exhibit poor intratumoral retention, and cause nonspecific toxicity [24, 25, 26]. These challenges underscore the urgent need for an integrated platform that combines robust piezocatalytic‐photothermal performance with tumor‐targeted delivery and controlled activation.

**SCHEME 1:**
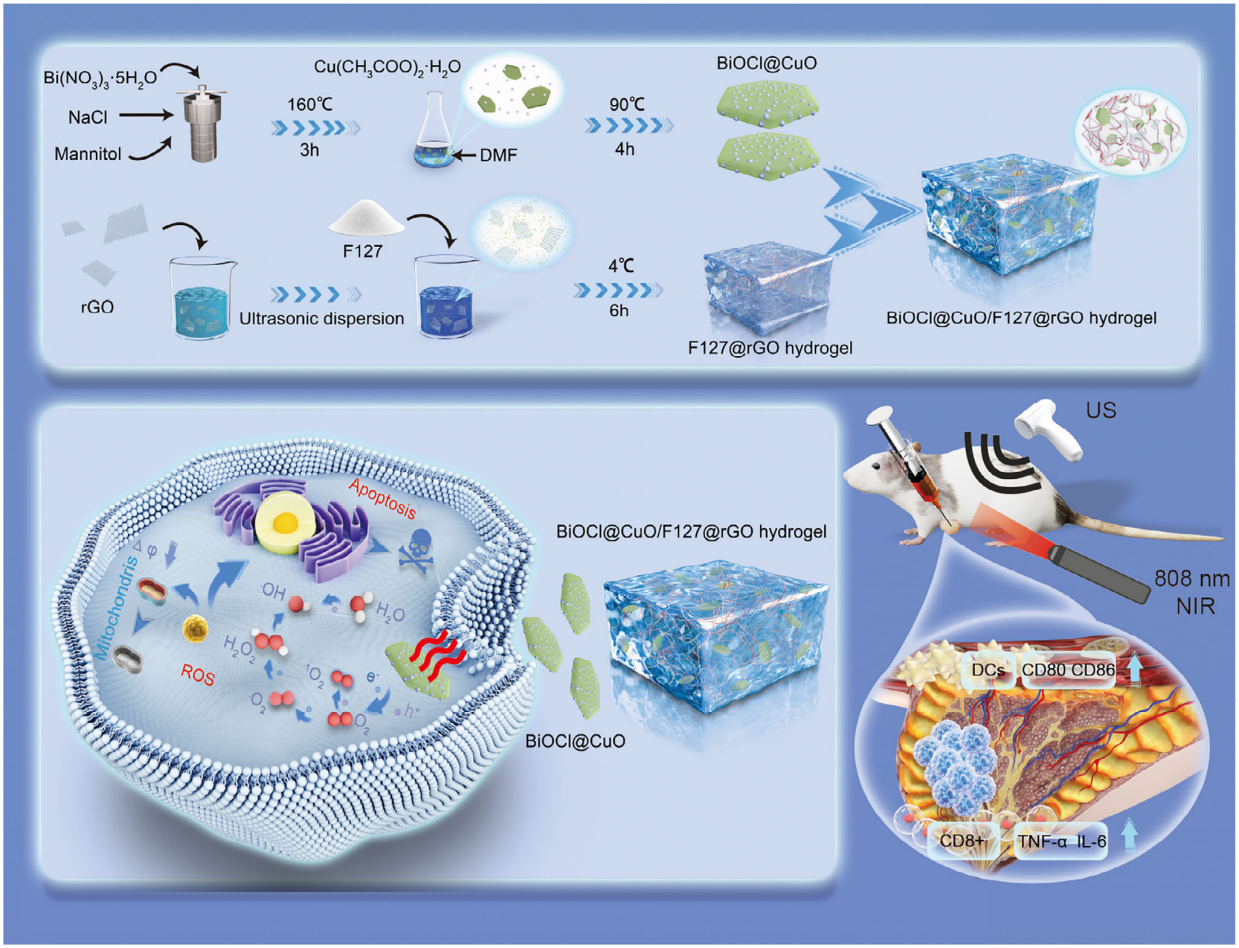
Schematic diagram of the synthesis of BiOCl@CuO/F127@rGO hydrogel and its application in breast cancer.

Herein, we designed a fundamentally novel heterostructured BiOCl@CuO nanosheet that addresses these limitations. Unlike prior systems, we leverage layered BiOCl with intrinsic piezoelectricity as the substrate and grow CuO in situ to create a heterojunction with an exceptionally strong built‐in electric field, achieving superior charge separation efficiency under US stimulation. Critically, BiOCl@CuO exhibits dual absorption in both US and NIR regions, enabling genuine synergistic piezocatalysis‐photothermal coupling rather than simple co‐delivery of separate agents. To overcome the delivery challenges that plague existing systems, we integrated these nanosheets within an injectable conductive thermosensitive F127@rGO hydrogel composed of Pluronic F127 (F127) and reduced graphene oxide (rGO). This platform provides distinct advantages over previous reports. Notably, F127 enables immediate sol‐gel transition at physiological temperature, forming a stable intratumoral depot that prevents nanoparticle dispersal and systemic toxicity [27]. Furthermore, rGO constructs a conductive network that suppresses electron‐hole recombination, directly amplifying piezocatalytic efficiency—a feature absent in non‐conductive hydrogel carriers [28]. Additionally, the hydrogel matrix maintains BiOCl@CuO in close proximity to tumor cells, ensuring continuous mechanical energy transfer and ROS delivery. This integrated BiOCl@CuO/F127@rGO hydrogel system thus represents a significant advance by combining an optimized heterojunction design with an intelligent delivery system that actively participates in the therapeutic mechanism, offering a promising approach for safe, efficient, and precise breast cancer treatment (Scheme 1).

## 2 Results and Discussion

**FIGURE 1:**
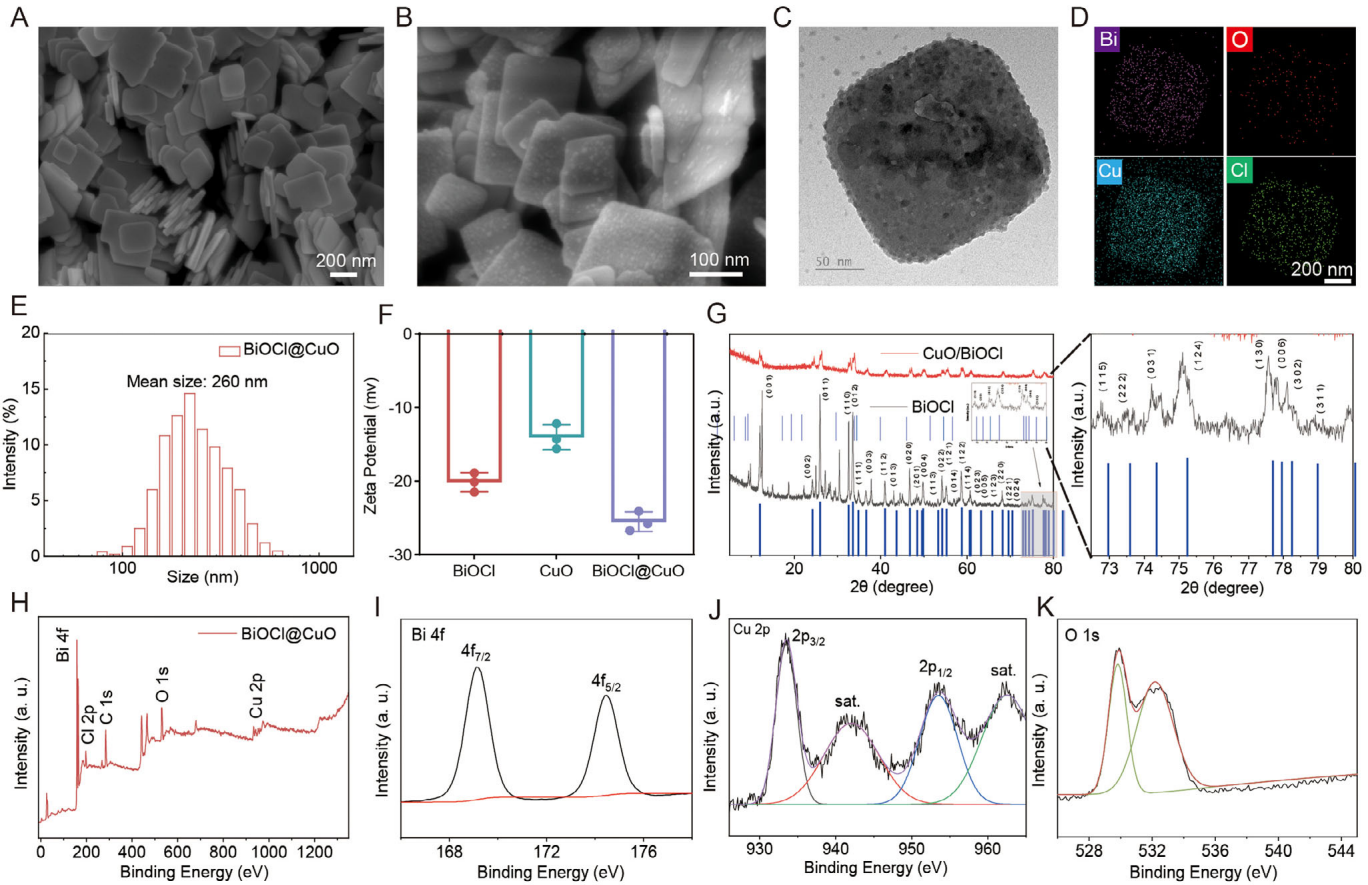
Characterization of BiOCl@CuO nanosheets. (A, B) SEM image of BiOCl (A) and BiOCl@CuO (B). (C) TEM image of BiOCl@CuO. (D) EDS mapping of a representative BiOCl@CuO. (E) Size distribution of the BiOCl@CuO. (F) Zeta potential of BiOCl, CuO, and BiOCl@CuO, respectively (n = 3). The data are presented as the means ± SD. (G) XRD pattern of BiOCl@CuO. (H) XPS spectrum of the BiOCl@CuO. (I) High‐resolution XPS spectrum of Bi 4f for the BiOCl@CuO. (J) High‐resolution XPS spectrum of Cu 2p for the BiOCl@CuO. (K) High‐resolution XPS spectrum of O 1s for the BiOCl@CuO.

BiOCl nanosheets were synthesized via the solvothermal method, after which CuO was in situ grown on BiOCl to form a composite heterostructure (BiOCl@CuO) following the published protocol [29]. Figure 1A, B shows the scanning electron microscopy (SEM) images of BiOCl and BiOCl@CuO, respectively, and the CuO was uniformly distributed on the nanosheets. The transmission electron microscopy (TEM) and X‐ray spectroscopy (EDS) mapping of Bi, O, Cl, and Cu, in Figure 1C, D, which further proved the successful synthesis of BiOCl@CuO. TEM and SEM analysis revealed these nanosheets had a size distribution of 100–400 nm. Dynamic light scattering (DLS) yielded an average hydrodynamic diameter of 260 ± 2.2 nm, representing the spherical equivalent of these 2D nanostructures in aqueous solution (Figure 1E). Figure 1F shows the zeta potential result values of BiOCl (−20 ± 2 mV), CuO (−15 ± 2 mV), and BiOCl@CuO (−25 ± 1.5 mV). The incorporation of CuO further enhances the surface charge of BiOCl@CuO, which could improve the stability and dispersibility. X‐ray diffraction (XRD) results (Figure 1G) show that the BiOCl@CuO nanosheets exhibited the characteristic peaks of both BiOCl and CuO simultaneously. This further confirmed the heterojunction between BiOCl and CuO. X‐ray photoelectron spectroscopy (XPS) results show the chemical state of the BiOCl@CuO heterojunction (Figure 1H–K). The Bi, O, Cl, and Cu were all present in the sample, and the atomic ratios of Bi:O:Cl and Cu:O were be 1:1:1 and 1:1, respectively, based on calculation. In BiOCl@CuO (Figure 1H), the spin orbital splitting photoelectrons of Bi (4f_5/2_) and Bi (4f_7/2_) shifted to 175.4 and 169.3 eV, indicating the strong interaction between CuO nanoparticles and BiOCl nanosheets at their interface (Figure 1I). Figure 1J shows the high‐resolution XPS spectrum of Cu 2p. The Cu 2p orbitals split into two main peaks, Cu 2p_1/2_ and Cu 2p_3/2_, with a separation of about 20 eV, which was typical for copper. Additionally, two distinct satellite peaks were presented at higher binding energies of the main peaks, further demonstrating the existence of Cu^2+^. In the O 1 s spectrum, two apparent peaks were found in which a low binding energy peak was related to lattice oxygen, such as Cu‐O and Bi‐O bonding, while a high binding energy peak was related to adsorbed oxygen, caused by the generated oxygen vacancies (Figure 1K) [30]. These results verified the formation of the BiOCl@CuO heterojunction.

**FIGURE 2:**
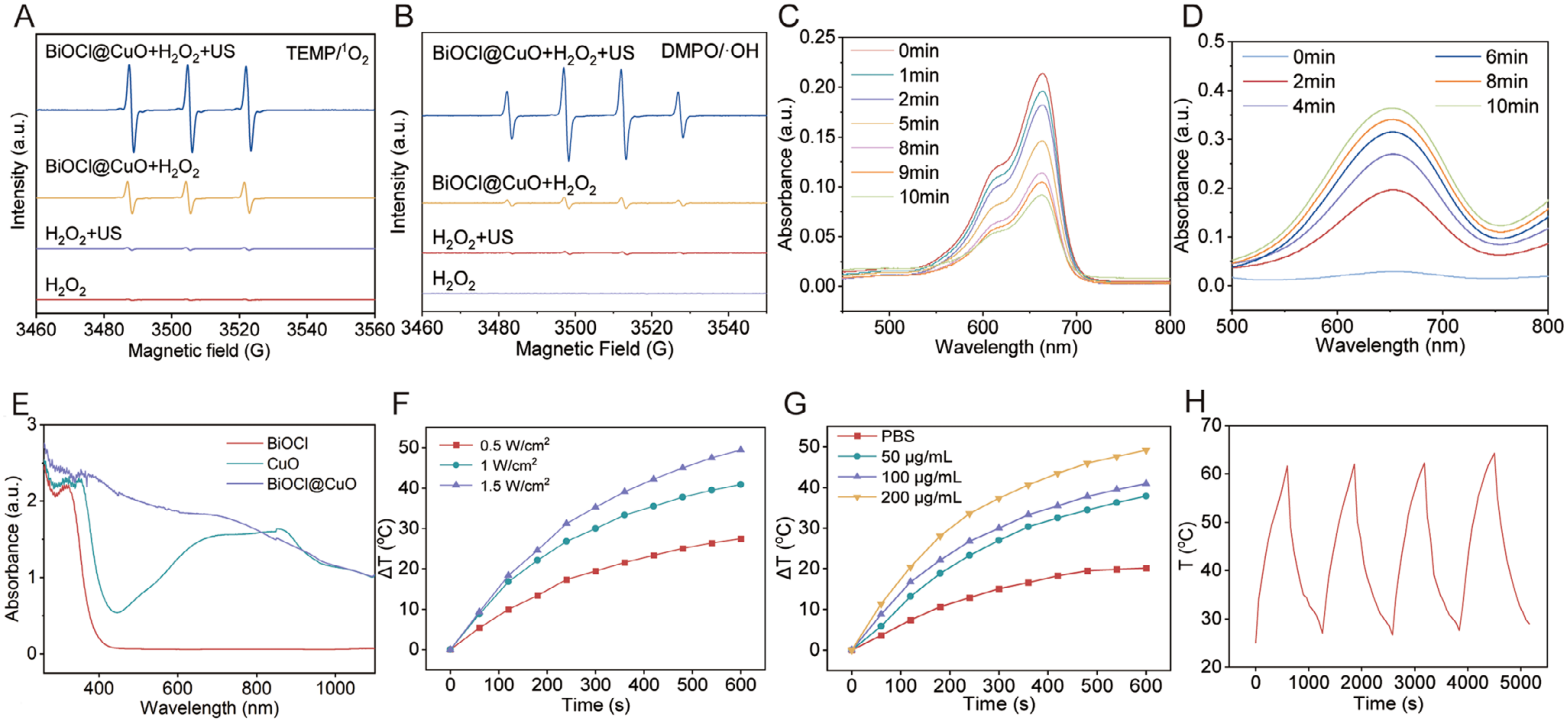
In vitro catalytic and photothermal performance of BiOCl@CuO nanosheets. (A) ESR spectra of ^1^O_2_ trapped by 2, 2, 6, 6‐tetramethylpiperidine (TEMP) with BiOCl@CuO nanosheets under ultrasound. (B) ESR spectra of ·OH trapped by 5, 5‐dimethyl‐1‐pyrrolidine N‐oxide (DMPO) with BiOCl@CuO nanosheets under ultrasound. (C) Absorbance of MB treated with BiOCl@CuO nanosheets under ultrasound at different times. (D) Absorbance of TMB oxidation‐treated with BiOCl@CuO nanosheets under ultrasound at different times. (E) The ultraviolet absorbance of BiOCl, CuO, and BiOCl@CuO nanosheets. (F) Temperature of BiOCl@CuO nanosheets under different light intensities. (G) Temperature of different concentrations of BiOCl@CuO nanosheets under light irritation. (H) Photothermal cycling test of BiOCl@CuO nanosheets.

Electron spin resonance (ESR) spectroscopy was utilized to test the generation of ROS under US. Figure A shows that H_2_O_2_ and H_2_O_2_+US group exhibited negligible ^1^O_2_ signal, and only a slightly enhanced signal in the BiOCl@ piezocatalytic and photothermal CuO+H_2_O_2_ group. In contrast, the BiOCl@CuO+H_2_O_2_ +US group exhibited a remarkable ^1^O_2_ signal increase (Figure 2A). As shown in Figure 2B, the BiOCl@CuO+H_2_O_2_ +US group also presented a distinct signal intensity of ·OH than that in H_2_O_2_, H_2_O_2_+US, and BiOCl@CuO+H_2_O_2_ groups. Collectively, BiOCl@CuO can effectively generate ROS under ultrasound. As shown in Figure 2C, the absorbance of methylene blue (MB) gradually decreased as treatment time increased from 0 to 10 min, indicating increased ·OH generated by BiOCl@CuO under US. Additionally, Figure 2D showed obvious absorbance from oxidized 3,3',5,5'‐Tetramethylbenzidine (TMB), which gradually increased with BiOCl@CuO under US and with treatment time from 0 to 10 min. These demonstrated the efficient ROS generation capacity of BiOCl@CuO under US. Figure 2E shows that BiOCl@CuO nanosheets exhibited stronger light absorption intensity in the wavelength range of 400–1000 nm compared to BiOCl and CuO. In addition, the temperature of the nanosheets elevated progressively under gradually increased NIR intensity (0.5, 1, and 1.5 W/cm^2^) (Figure 2F). Under NIR irradiation, PBS alone exhibited a temperature increase of approximately 20°C. BiOCl@CuO nanosheets demonstrated concentration‐dependent photothermal effects, producing progressively greater temperature rises with increasing concentrations (50, 100, 200 µg/mL), achieving a ΔT of 50°C at 200 µg/mL (Figure 2G). These BiOCl@CuO nanosheets exhibited excellent photothermal cycling stability (Figure 2H).

**FIGURE 3:**
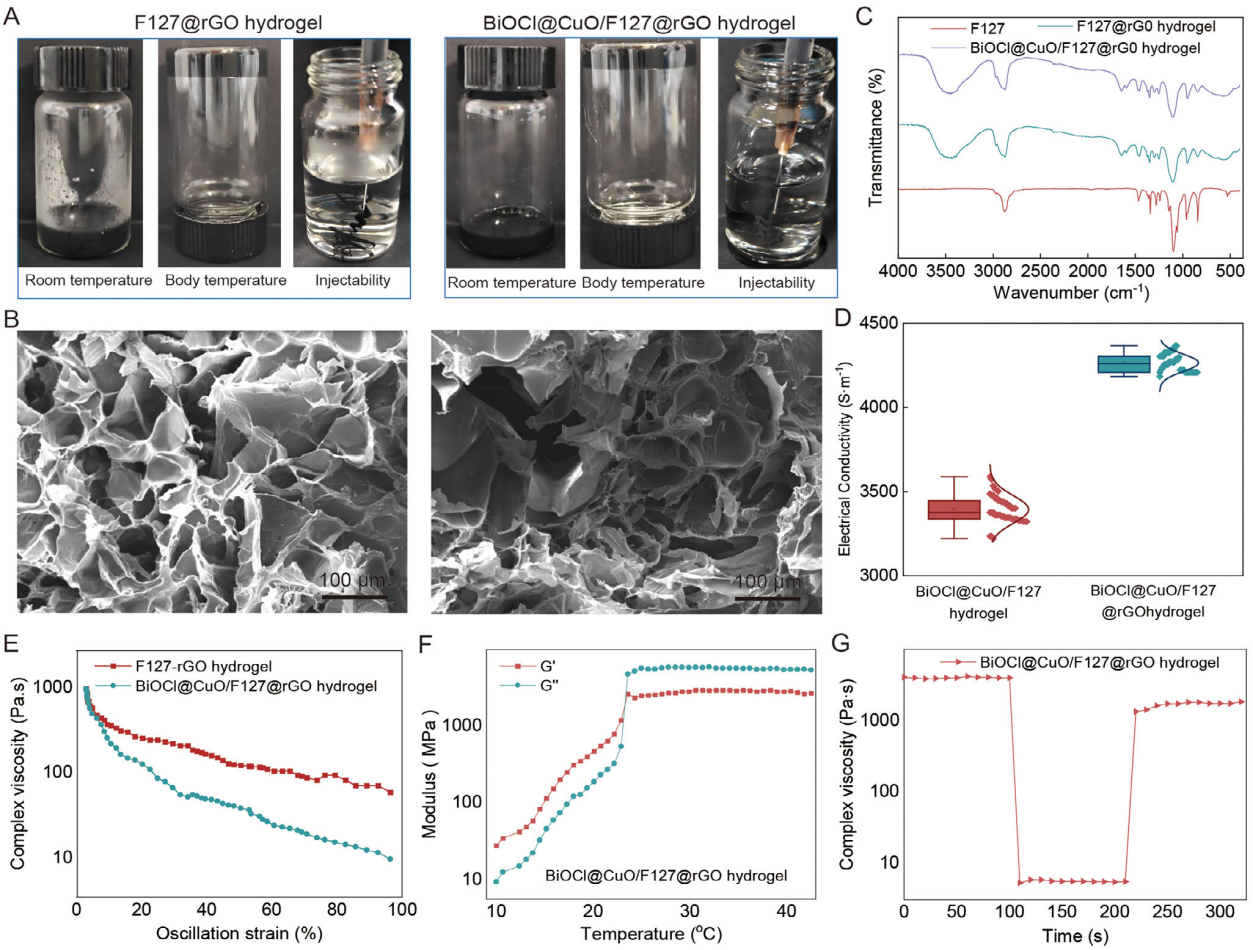
Characterization of BiOCl@CuO/F127@rGO hydrogel. (A) Photographs of BiOCl@CuO/F127@rGO hydrogel. (B) SEM image of FHG hydrogel. (C) FTIR spectra of BiOCl@CuO/F127@rGO hydrogel. (D) Electrical conductivity of BiOCl@CuO/F127 and BiOCl@CuO/F127@rGO hydrogels. (E) Oscillation strain test of BiOCl@CuO/F127@rGO hydrogel. (F) Dynamic rheological test of BiOCl@CuO/F127@rGO hydrogel. The vertical coordinate shows the values of storage modulus (G')/loss modulus (G''). G) Step strain rheological test of BiOCl@CuO/F127@rGO hydrogel.

Then the thermosensitive hydrogel was synthesized with F127. Subsequently, rGO and BiOCl@CuO nanosheets were introduced to be added into the F127 hydrogel through simple mixing to form the final hydrogel (BiOCl@CuO/F127@rGO). As shown in Figure 3A, the hydrogel remained in a flowing state at room temperature and rapidly gelled at body temperature before and after the addition of BiOCl@CuO, and the BiOCl@CuO/F127@rGO hydrogel exhibited great injectability. SEM images in Figure 3B demonstrate the successful incorporation of BiOCl@CuO, with the hydrogel structure remaining unchanged. Figure 3C shows the Fourier transform infrared spectroscopy (FTIR) of F127, F127@rGO, and BiOCl@CuO/F127@rGO hydrogels. The characteristic peaks of F127 and F127@rGO appeared in BiOCl@CuO/F127@rGO, indicating the successful synthesis of the composite hydrogel. The BiOCl@CuO/F127 hydrogel exhibits an electrical conductivity of around 3500 S·m^−1^. When add the rGO into the BiOCl@CuO/F127 hydrogel, the electrical conductivity increased to approximately 4250 S·m^−1^ (Figure 3D). The result indicates that the incorporation of rGO significantly improved the conductivity of the hydrogel. This conductive network facilitates rapid electron transfer, suppressing electron‐hole recombination in the BiOCl@CuO heterostructure and thereby amplifying piezocatalytic ROS generation efficiency—a critical enhancement absent in conventional insulating hydrogel carriers.

To further evaluate the mechanical reinforcement provided by rGO, comprehensive rheological assays were performed (Figure S1 and Table S1). Frequency sweep results (Figure S1C) show that G' consistently exceeds G'' from 0.1 to 10 Hz at 37°C, demonstrating a robust, elastic‐dominated “strong gel” state. The mechanical stability of this viscoelastic network is further confirmed by time‐dependent oscillatory measurements (Figure S1A). Notably, the complex viscosity exhibits a sharp decreasing trend as the shear rate increases (Figure S1B). A typical shear‐thinning behavior is quantified in Table S1 (dropping from 0.8 ± 0.1 to 0.15 ± 0.05 Pa·s). Consistent with these findings, the oscillatory strain experiment (Figure 3E) shows that the hydrogel processes excellent resilience under increasing strain. This suggests that the hydrogel processed excellent injectability. Additionally, the hydrogel demonstrated favorable degradation and swelling properties (Figure S2), and a sustained release profile of Cu^2^^+^ ions was observed over 72 h (Figure S3). Figure 3F showed the changes of G' and G'' of BiOCl@CuO/F127@rGO hydrogel with temperature. When the temperature gradually increased from 10°C to 23°C, the change trends of G' and G'' values of BiOCl@CuO/F127@rGO hydrogel were obvious. As the temperature approached the range of 23°C ‐ 24°C, the storage modulus began to increase rapidly, while the loss G'' also increased, but the increase range was relatively small. When the temperature reached a certain level, the value of G' exceeded G'', and the gap gradually widened, which indicated that the material gradually changed from a viscous‐dominated state to an elastic‐dominated gel state. The result indicated that BiOCl@CuO/F127@rGO hydrogels had great temperature‐sensitive properties. Figure 3G shows that the complex viscosity remains at a relatively high level at the very beginning. As time approached 100 s, the complex viscosity dropped sharply to a low value and stayed at this low level until about 200 s. Subsequently, from 200 s onward, it returned to a high level and maintained stability. This trend indicates that the BiOCl@CuO/F127@rGO hydrogel exhibits shear‐thinning and self‐healing properties.

To clarify the specific contributions of the hydrogel components to the therapeutic effect, ROS generation and photothermal conversion efficiency were systematically evaluated (Figure S4). ESR spectra revealed that the F127@rGO matrix alone produced no detectable ^1^O_2_ signals under US, whereas the incorporation of BiOCl@CuO nanostructures resulted in a robust 1:1:1 triplet signal, identifying the heterojunction as the primary sonosensitizer. Furthermore, the photothermal conversion capability was quantified (Figure S5). The BiOCl@CuO/F127@rGO hydrogel exhibited a rapid temperature rise to 67°C within 600 s. By linear fitting of the cooling stage, the system time constant (τ_s_) was found to be 215.05s, yielding a photothermal conversion efficiency (η) of 32.8%. These integrated features collectively established a robust platform for synergistic piezo‐photothermal therapy, with comprehensive parameters detailed in Table S1.

**FIGURE 4:**
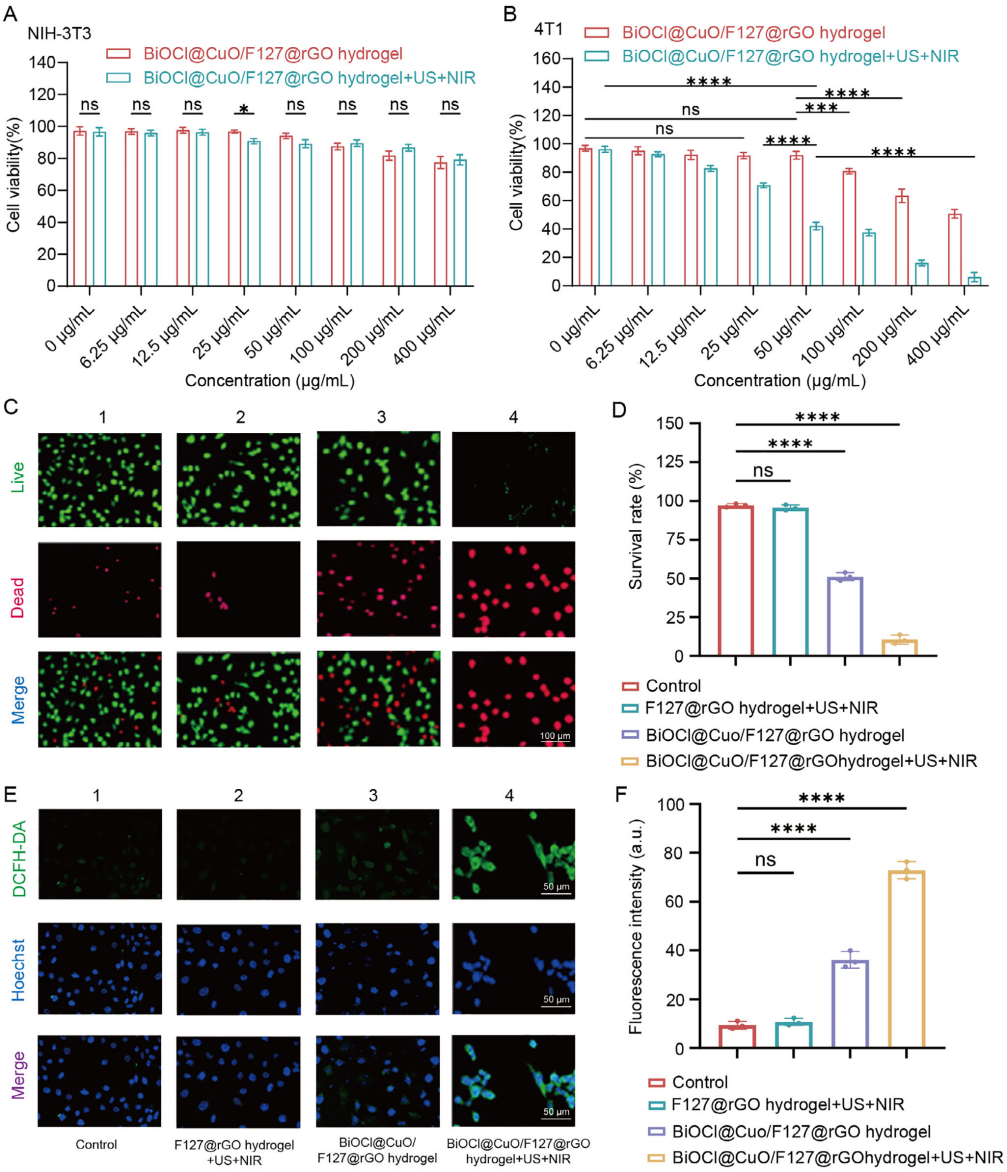
In vitro cancer therapeutic effect of BiOCl@CuO/F127@rGO hydrogel under US and NIR irradiation. (A) Cell viability of NIH‐3T3 cells after treatment with BiOCl@CuO/F127@rGO and BiOCl@CuO/F127@rGO+US+NIR (n = 3). (B) Cell viability of 4T1 cells after treatment with BiOCl@CuO/F127@rGO and BiOCl@CuO/F127@rGO+US+NIR (n = 3). (C) Live/Dead staining results of 4T1 cells after treatment with different groups. Cells without any treatment as the control group. Living and dead cells were stained green and red, respectively. (D) The corresponding statistical result showing the percentage of live cells (n = 3). (E) Fluorescence images of 4T1 cells after treatment with different groups (n = 3). Cells without any treatment as the control group. Nuclei were stained blue. (F) The corresponding statistical results showing the relative mean fluorescence intensity in E) (n = 3). The data are presented as the means ± SD. The p‐values were calculated via one‐way analysis of variance (ANOVA), ns>0.05, ^**^ *p* < 0.01, ^***^ *p* < 0.001, and ^****^ *p* < 0.0001.

To further explore the therapeutic efficacy of the BiOCl@CuO/F127@rGO hydrogel against cancer under US and NIR irradiation, a series of experiments was conducted. The cytotoxicity of the BiOCl@CuO/F127@rGO hydrogel toward normal cells was assessed using the Cell Counting Kit‐8 (CCK‐8) assay. As shown in Figure 4A, following 24 h after the treatment of BiOCl@CuO/F127@rGO hydrogel under US and NIR. When the concentration of BiOCl nanosheets was below 12.5 µg/mL, the survival rate of mouse NIH‐3T3 fibroblasts was maintained over 95%, exhibiting no statistically significant difference compared to the control group cells. Extended biocompatibility studies at 48 and 72 h (Figure S6) demonstrated that even at therapeutic concentrations (50 µg/mL), NIH‐3T3 cell viability remained >90% after 72 h incubation with BiOCl@CuO/F127@rGO, confirming excellent material safety. The cell survival rate in the BiOCl@CuO/F127@rGO+US+NIR group decreases slightly with the concentration of BiOCl exceeds 12.5 µg/mL. This observation confirmed that the BiOCl@CuO/F127@rGO hydrogel possesses excellent cytocompatibility with normal cells and does not induce significant cytotoxicity under US and NIR irradiation.

Figure 4B presents 4T1 cell viability after 24 h exposed to BiOCl@CuO/F127@rGO hydrogel at different concentrations of BiOCl nanosheets (0‐400 µg/mL) with and without ultrasound and NIR treatment. When the concentrations of BiOCl nanosheets are below 25 µg/mL, there is no significant difference in cell viability between the BiOCl@CuO/F127@rGO and the BiOCl@CuO/F127@rGO+US+NIR group. Similar 48 and 72 h studies on 4T1 cells (Figure S6) showed minimal dark toxicity, with viability >80% at 50 µg/mL after 72 h, validating the hydrogel's biocompatibility with tumor cells prior to activation. Importantly, beyond this threshold, the BiOCl@CuO/F127@rGO hydrogel+US+NIR group exhibited concentration‐dependent cytotoxicity enhancement, achieving about 50% cell reduction at 50 µg/mL and 90% reduction at 400 µg/mL, demonstrating significantly reduced cell viability compared to the BiOCl@CuO/F127@rGO control group. These significant differences indicate that under US and NIR irradiation, the BiOCl@CuO/F127@rGO hydrogel exhibits enhanced anti‐tumor activity. Live/Dead staining results (Figure 4C, D) provided visual confirmation of therapeutic efficacy, revealing that the BiOCl@CuO/F127@rGO+US+NIR group exhibited a dramatic reduction in cell survival lower than 20%, demonstrating potent tumor cell ablation. In stark contrast, the Control and F127@rGO groups showed minimal cytotoxicity, with higher than 90% survival, while the BiOCl@CuO/F127@rGO alone group achieved moderate killing with about 50% cell survival, likely reflecting the intrinsic dark toxicity of CuO nanoparticles in high concentration. Additionally, 2,7‐dichlorofluorescein diacetate (DCFH‐DA) was employed to investigate the changes in intracellular ROS levels across different groups (Figure 4E, F). Among all groups, the BiOCl@CuO/F127@rGO+US+NIR group displayed the highest intracellular ROS levels, with a 7‐fold increase in fluorescence intensity compared to the control. Collectively, these results demonstrated that the BiOCl@CuO/F127@rGO hydrogel under US and NIR can notably promote the generation of free radicals, which in turn induce cellular oxidative stress and thereby mediate tumor cell death.

**FIGURE 5:**
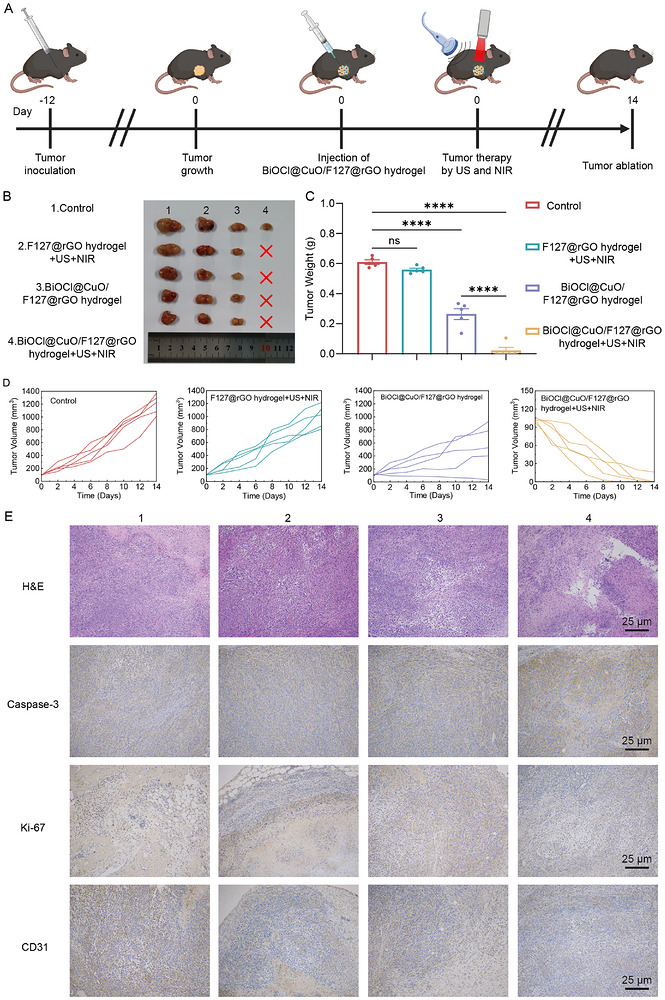
In vivo antitumor effects on in situ breast cancer. (A) Schematic illustration of the time schedule for the cancer treatment. (B) Photographs of excised tumor tissues at the end of the therapeutic period (n = 5). (C) Mean tumor weights measured after excision on day 14 (n = 5). (D) Representative tumor growth curves of mice from different groups over the 14‐day therapeutic period (n = 5). (E) H&E staining; and IHC staining for Caspase‐3, Ki‐67, and CD31 in tumor tissues from different groups. The data are presented as the means ± SD. The p‐values were calculated via one‐way analysis of variance (ANOVA), ns>0.05 and ^****^ *p* < 0.0001.

To assess the in vivo antitumor efficacy of the BiOCl@CuO/F127@rGO hydrogel, we established a mouse model of breast cancer, as shown in Figure 5A. On day 12 post‐inoculation, mice were randomly divided into four treatment groups: control, F127@rGO hydrogel+US+NIR, BiOCl@CuO/F127@rGO hydrogel, and BiOCl@CuO/F127@rGO hydrogel+US+NIR. Following 14 days of treatment, quantitative analysis exhibited that the BiOCl@CuO/F127@rGO hydrogel+US+NIR group exerted more potent tumor growth inhibitory effects relative to the other groups (Figure 5B). As shown in Figure 5C, D, tumor weights and volumes in the BiOCl@CuO/F127@rGO hydrogel+US+NIR group were markedly decreased. Notably, exclusive tumor regression was observed in the BiOCl@CuO/F127@rGO hydrogel+US+NIR group, which achieved an 90% tumor remission rate (Table S2)—indicating not merely tumor growth arrest, but also genuine reduction in tumor burden.

Histopathological analysis using Hematoxylin and Eosin (H&E) staining validated a significant loss of malignant cells in the BiOCl@CuO/F127@rGO hydrogel+US+NIR group (Figure 5E). Immunohistochemical analysis reveals significantly upregulated Caspase‐3 expression in the BiOCl@CuO/F127@rGO+US+NIR group versus controls, confirming robust activation of tumor apoptotic pathways. Concurrently, the decrease in the expression of Ki‐67 and CD31 demonstrates potent inhibition of tumor growth and angiogenesis. These results confirmed that the treatment triggers multiple coordinated anti‐tumor mechanisms—apoptotic induction, proliferative arrest, and vascular normalization—while the hydrogel platform ensures sustained local drug retention and minimizes systemic toxicity. This comprehensive anti‐tumor response, achieved through a single injectable formulation, positions the BiOCl@CuO/F127@rGO hydrogel as a promising translational strategy for precision breast cancer therapy.

**FIGURE 6:**
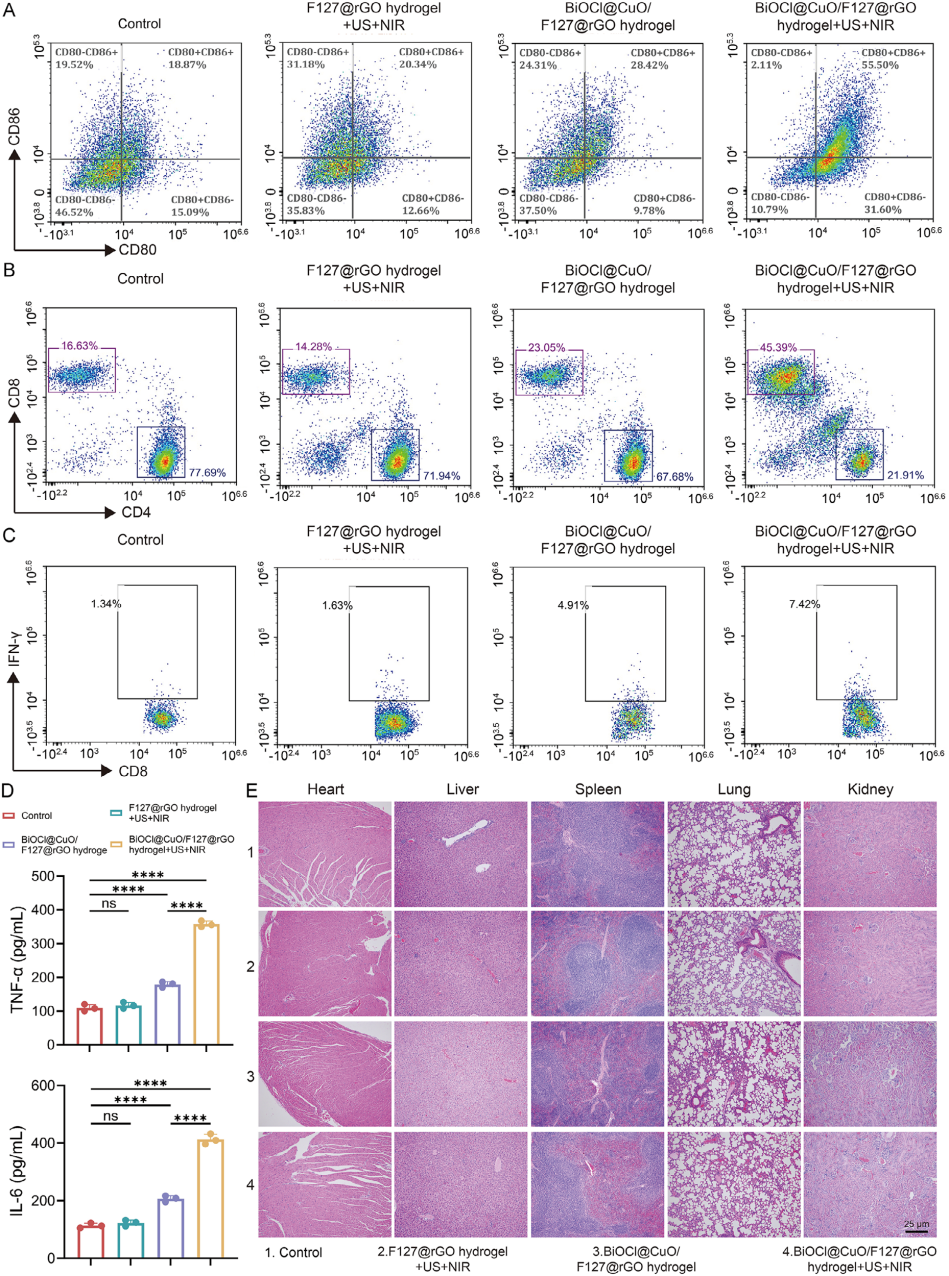
In vivo local immune activation in orthotopic breast cancer. (A) Representative FACS analysis images of CD86+, CD80+, and DCs after treatment. (B) Representative FACS analysis images of the proportion of CD4+ and CD8+ T cells in T cells after treatment. (C) Representative FACS analysis images of IFN‐γ+ CD8+ T cells after treatment. (D) Secretion levels of cytokines TNF‐α and IL‐6 in the serum by ELISA (n = 3). (E) H&E staining of the heart, breast, spleen, lung, and kidney of mice from different groups. The data are presented as the means ± SD. The p‐values were calculated via one‐way analysis of variance (ANOVA), ns>0.05 and ^****^ *p* < 0.0001.

To systematically explore the immunomodulatory effects of BiOCl@CuO/F127@rGO hydrogel under US and NIR irradiation, we further assessed the activation of tumor‐infiltrating immune cells. Flow cytometry confirmed pronounced upregulation of dendritic cell (DCs) co‐stimulatory molecules CD80 and CD86 by 3‐fold in tumors treated with BiOCl@CuO/F127@rGO hydrogel under US and NIR against control (Figure 6A), demonstrating potent local antigen‐presenting cell activation. This combined treatment also notably elevated both the proportion of CD8+ T cells in the infiltrate by 2.7‐fold and their IFN‐γ secretion by 5‐fold (Figure 6B, C), suggesting robust activation of cytotoxic CD8+ T cell responses. Furthermore, ELISA analysis revealed significantly elevated levels of the pro‐inflammatory cytokines TNF‐α and IL‐6 in the tumor microenvironment (Figure 6D), consistent with the establishment of a local inflammatory milieu driven by ROS‐mediated signaling pathways [31]. To confirm that the observed immune activation was triggered by immunogenic cell death, we measured High Mobility Group Protein 1 (HMGB1) release, a key damage‐associated molecular pattern. ELISA analysis revealed a 3‐fold increase in HMGB1 levels in the tumor microenvironment following BiOCl@CuO/F127@rGO+US+NIR treatment (Figure S7), providing direct evidence of local ICD induction. The above results indicate the successful priming of local type I and type II immune responses within the tumor microenvironment [32, 33, 34, 35, 36]. Critically, comprehensive safety assessments via histological analyses of major organs revealed no signs of pathological alterations or inflammatory infiltrates (Figure 6E). Body weight monitoring showed no significant loss across treatment groups over 14 days (Figure S8), while complete serum biochemistry panels (Figure S9) and blood routine panels (Figure S10) remained within normal ranges, indicating absent systemic toxicity. These results confirmed the favorable in vivo biocompatibility of the BiOCl@CuO/F127@rGO hydrogel. This BiOCl@CuO/F127@rGO hydrogel‐mediated piezocatalytic and photothermal combination therapy illustrates that it exerts multimodal therapeutic effects by directly inducing immunogenic tumor cell apoptosis via piezoelectric catalysis and hyperthermia, leading to local activation of DCs and CD8+ effector cytotoxic T cells within the tumor microenvironment and establishing a pro‐inflammatory tumor niche that favors anti‐tumor immune priming. The synergistic integration of localized piezocatalytic and photothermal effects establishes the BiOCl@CuO/F127@rGO hydrogel as a promising precision therapeutic platform for breast cancer, with the dual advantages of targeted tumor ablation and local immune priming. While our data demonstrate robust local DC maturation, CD8+ T cell activation, and pro‐inflammatory cytokine release within the tumor, we acknowledge that the induction of systemic anti‐tumor immunity and long‐term immunological memory requires further validation through bilateral tumor models and tumor rechallenge studies, important directions for future research. The current study is focused on characterizing the local immune‐priming effects of this therapeutic platform.

## 3 Conclusions

In summary, we overcome the intrinsic constraints of PCT by developing a multifunctional therapeutic system based on BiOCl@CuO heterostructured nanosheets embedded within an injectable, conductive, thermosensitive hydrogel formulated with F127 and rGO. Under US irradiation, it generates a strong interfacial electric field that efficiently suppresses electron–hole recombination and accelerates the generation of tumor‐lethal ROS. Concurrently, NIR irradiation activates the photothermal conversion capability of BiOCl@CuO, which not only elevates the intratumoral temperature to amplify ROS‐mediated cytotoxicity but also facilitates PTT, thereby realizing synergistic piezocatalytic‐photothermal treatment. In vivo, tumors treated with the combination of US and NIR irradiation exhibited pronounced regression, accompanied by suppressed tumor cell proliferation and angiogenesis, as well as activation of apoptotic and immunogenic cell death pathways. Importantly, the entire system retained excellent biocompatibility. This study establishes a precise, minimally invasive, and spatiotemporally precise therapeutic strategy that innovatively integrates piezocatalysis with photothermal conversion. By surmounting the limitations of PCT and leveraging multimodal therapeutic synergism, this system provides a promising and effective approach for breast cancer treatment.

## 4 Experimental Section

### 4.1 Materials

Bismuth nitrate pentahydrate (Bi(NO_3_)_3_·5H_2_O, 99.9%), mannitol (99%), sodium chloride (NaCl, 99.5%), copper acetate monohydrate (Cu(CH_3_COO)_2_·H_2_O, 99%), N, N‐dimethylformamide (DMF, 99.8%, anhydrous), and anhydrous ethanol (99.9%) were obtained from Aladdin (China). Pluronic F127 (biomedical grade), rGO (purity >95%, single layer), tetramethylbenzidine (TMB, 99%), methylene blue (MB, 98%), and dimethyl sulfoxide (DMSO, 99.9%, anhydrous) were purchased from Sigma‐Aldrich (USA). Phosphate‐buffered saline (PBS), Cell Counting Kit‐8 (CCK‐8), and Calcein/PI Cell Viability/Cytotoxicity Assay Kit were purchased from Beyotime Biotechnology (China). A protease inhibitor cocktail was purchased from Thermo Scientific (USA). All fluorescently labelled antibodies used in the experiments were purchased from Biolegend (USA). The ELISA Kit of TNF‐α and IL‐6 was purchased from Thermo Scientific (USA).

### 4.2 Characterization

SEM images were obtained by a field‐emission Magellan 400 microscope (FEI Company, US). TEM images were acquired by a JEM‐2100F electron microscope (200 kV). The Zetasizer Nanoseries (zeta Plus, Brookhaven Instruments Corporation) was utilized to analyze zeta potential. XRD analysis was acquired on a Rigaku D/MAX‐2200 PC XRD system. XPS analysis was carried out by ESCAlab250 (Thermal Scientific, US). (ESR) Signals were recorded on an ESR spectrometer (JEOL‐FA200, JEOL, Japan). UV–vis absorption spectra were recorded on a UV‐3600 Shimadzu UV–vis spectrometer. Temperature changes under 808 nm NIR irradiation were monitored in real‐time using a FLIR A655sc infrared thermal imager. Confocal images were acquired on a CarlZeiss LSM900 (CLSM. Flow cytometry analysis was performed on a BD LSRFortessa flow cytometer.

### 4.3 Preparation of the BiOCl@CuO

BiOCl nanosheets were fabricated via a one‐pot solvothermal approach. Briefly, 0.486 g of bismuth nitrate pentahydrate (Bi(NO_3_)_3_·5H_2_O) was dissolved in 25 mL of a 0.1 mol/L mannitol solution under continuous stirring for 30 min. Subsequently, 5 mL of a saturated NaCl solution was added dropwise to the above mixture, followed by further stirring for an additional 30 min. The above solution was then transferred into a Teflon‐lined stainless‐steel autoclave and reacted at 160°C for 3 h. Upon completion of the solvothermal reaction, the precipitate formed was collected and washed alternately with ethanol and deionized water, with each solvent used for 3 washing cycles. The washed product was dried at 60°C for 4 h and ground into a fine powder for subsequent use. For the fabrication of BiOCl@CuO, 0.65 g of the as‐prepared BiOCl and 0.05 g of Cu(CH_3_COO)_2_·H_2_O were added into an Erlenmeyer flask containing 50.0 mL of DMF. The mixture was stirred continuously for 15 min, after which the Erlenmeyer flask was transferred into a constant‐temperature water bath set at 90°C for prolonged stirring (4 h). Once the solution cooled to room temperature, the BiOCl@CuO composite was collected by centrifugation at 9000 rpm for 5 min. The collected composite was rinsed with deionized water for 3 cycles and dried at 60°C for subsequent experiments.

### 4.4 Preparation of the BiOCl@CuO/F127@rGO Hydrogel

First, rGO was dispersed in 8 mL of deionized water at a concentration of 5 mg/mL, and uniform dispersion was achieved via ultrasonic treatment. Subsequently, 1.9 g of F127 powder was added to the above rGO dispersion, followed by magnetic stirring at 4°C for 4 h to obtain an F127@rGO composite solution. Finally, the previously prepared BiOCl@CuO were added to the F127@rGO solution, and the BiOCl@CuO/F127@rGO solution was stored at 4°C overnight.

### 4.5 Chemodynamic Performance

To detect the generation of hydroxyl radicals (•OH) during the chemodynamic process of BiOCl@CuO in the presence of H_2_O_2_, MB and TMB were employed as the chromogenic probe. Specifically, a 200 µL suspension of BiOCl@CuO (7 mg/mL) was added to a 7 mL MB solution (5 mg/mL). Subsequently, 10 µL of 30% H_2_O_2_ solution was added into the mixture. The mixture was subjected to US irradiation at different times (0, 1, 2, 5, 8, 9, and 10 min), followed by centrifugation and analysis of the supernatant using UV–vis absorption spectroscopy. As for the detection using TMB, 200 µL of BiOCl@CuO (7 mg/mL) was added to a 7 mL TMB solution (5 mg/mL). Subsequently, 10 µL of 30% H_2_O_2_ solution was added into the mixture. The mixture was subjected to US irradiation at different times (0, 2, 4, 6, 8, and 10 min), followed by centrifugation and analysis of the supernatant using UV–vis absorption spectroscopy.

### 4.6 Photothermal Performance of BiOCl@CuO

The synthesized BiOCl@CuO was dispersed in deionized water to prepare a suspension with a concentration of 0.2 mg/mL. A 0.2 mL of this suspension was transferred into a 1.5 mL sterile conical centrifugal tube. For photothermal performance evaluation, an 808 nm NIR was used to irradiate the suspension, and the real‐time temperature variation of the suspension was recorded using a thermal imager (FLIR A655sc, USA) during the irradiation process. To investigate the effect of BiOCl@CuO concentration on its photothermal performance, the concentration of BiOCl@CuO was adjusted to 0, 50, 100, and 200 µg/mL, while other experimental conditions were kept constant. For each concentration, the temperature‐rise curves of the suspension were recorded. Similarly, to clarify the influence of laser power density on the photothermal effect, the concentration of BiOCl@CuO was maintained constant at 200 µg/mL, while the laser power density was varied (0.5, 1.0, and 1.5 W/cm^2^). Under each power density, the temperature evolution of the suspension was monitored to evaluate the photothermal response. The four successive heating–cooling curves of BiOCl@CuO with a concentration of 200 µg/mL and a power density of 1 W/cm^2^ were used to test the photothermal stability. The temperatures described above were recorded every 30 s.

### 4.7 Rheological Test

Rheological characterizations of the as‐prepared BiOCl@CuO/F127@rGO hydrogel were performed using a Physica MCR 301 rheometer (Anton Paar GmbH, Austria). To minimize water evaporation‐induced volume shrinkage, the hydrogel sample was carefully deposited onto the lower plate of the rheometer, and a thin layer of high‐purity low‐density mineral oil was applied around the hydrogel edge to seal the sample. For dynamic strain scanning tests, the strain range was applied from 0.1% to 100%. The temperature ramped from 10 to 42°C at a heating rate of 1°C/min. The frequency sweep, amplitude sweep, and temperature sweep measurements were performed within the linear viscoelastic region.

### 4.8 Cell Lines

Both the 4T1 (RRID: CVCL_0125) and NIH‐3T3 (RRID: CVCL_0594) cell lines used in this study were purchased from Procell Biotechnology Co., Ltd. (Wuhan, China), and STR authentication confirmed that these cell lines were free of cross‐contamination.

### 4.9 In Vitro Cell Viability Assay

To evaluate cytocompatibility, BiOCl@CuO/F127@rGO hydrogel extracts were prepared by incubating 0.2 g of hydrogel in 1 mL culture medium for 24 h at 37°C. NIH‐3T3 and 4T1 cells were seeded in 96‐well plates at 8 × 10^3^ cells per well and treated with the extracts at concentrations of 0, 6.25, 12.5, 25, 50, 100, 200, and 400 µg/ml. Cells were divided into two groups: one receiving ultrasound treatment (1.0 MHz, 1.5 W/cm^2^, 5 min) and one without ultrasound. After 24 h incubation, cell viability was assessed by CCK‐8 assay: 10 µL CCK‐8 solution was added to each well and incubated for 1–2 h at 37°C. Absorbance at 450 nm was measured using a microplate reader (Multiscan MK3, Thermo, USA). Cell viability (%) was calculated as: [(OD_treated_—OD_blank_)/(OD_control_—OD_blank_)] × 100%.

### 4.10 Live/Dead Staining

For Live/Dead staining, 8×103 of 4T1 cells were cultured in 96‐well culture plates and divided into four groups: 1) Control; 2) F127@rGO hydrogel+US+NIR; 3) BiOCl@CuO/F127@rGO hydrogel; 4) BiOCl@CuO/F127@rGO hydrogel+US+NIR. Groups 2 and 4 received immediate ultrasound (1.0 MHz, 1.5 W/cm^2^, 5 min) followed by NIR irradiation (808 nm, 0.5 W/cm^2^, 20 min). 300 µL of neurobasal medium with calcein‐AM (0.5 µM) and PI (3 µM) was added after culturing for 24 h. Subsequently, the samples were incubated at 37°C for 30 min. The CLSM was used to observe the samples. The software Image J was used to quantify the quantity of live and dead cells in three selected images from each group to determine the survival rate.

### 4.11 Detection of Intracellular Levels ROS

1 × 10^4^ of 4T1 cells were cultured in 48‐well culture plates and divided into four groups: 1) Control; 2) F127@rGO hydrogel+US+NIR;3) BiOCl@CuO/F127@rGO hydrogel; 4) BiOCl@CuO/F127@rGO hydrogel+US+NIR. Groups 2 and 4 received immediate ultrasound (1.0 MHz, 1.5 W/cm^2^, 5 min) followed by NIR irradiation (808 nm, 0.5 W/cm^2^, 20 min). After incubation for 24 h, the intracellular ROS levels were detected by DCFH‐DA. The fluorescence was observed using the CLSM, and the relative fluorescence intensity was quantified using ImageJ software from three selected images from each group.

### 4.12 Animal Models

Female C57BL/6 mice (6–8 week old, 18–20 g) were obtained from Charles River Laboratories (China). Mice were housed and fed sterile food with standard mice nutritional formula and sterile water in the isolated cages of a 12 h light/dark cycle environment. 4T1 mouse breast cancer cells were used for model establishment. Mice were anesthetized via inhalation of 2% isoflurane mixed with medical oxygen. Using a 29‐gauge insulin syringe, 50 µL of the cell suspension (containing 2 × 10^6^ cells) was injected into the fourth mammary fat pad of each mouse for orthotopic implantation. Post‐injection, mice were placed on a heating pad until full recovery from anesthesia. The study design, experimental procedures, husbandry and management, sample size, assignment of animals to experimental groups, and experimental results of the animal experiments in this study adhered to ARRIVE guidelines.

### 4.13 In Vivo Tumor Treatment and Biosafety Studies

For 4T1 tumor therapy, when the tumor size reached approximately 100 mm^3^ (designed as day 0), the mice were randomly divided into four groups (n = 5): 1) Control; 2) F127@rGO hydrogel+US+NIR; 3) BiOCl@CuO/F127@rGO hydrogel; 4) BiOCl@CuO/F127@rGO hydrogel+US+NIR. On day 0, 100 µL of the respective hydrogel was injected intratumorally into mice in groups 2, 3, and 4. Groups 2 and 4 received immediate ultrasound (1.0 MHz, 1.5 W/cm^2^, 5 min) followed by NIR irradiation (808 nm, 0.5 W/cm^2^, 20 min). Tumor volumes and body weights were recorded every 2 days. Tumor volume was calculated using the formula volume = (tumor length) × (tumor width)^2^ × 0.5. After 14 days of treatment, the mice were euthanized, and the tumors were weighed and photographed after dissection to evaluate the treatment effects. The tumor sections from each group were further analyzed via H&E staining and IHC staining for Caspase‐3, Ki‐67, and CD31. Major organs (heart, liver, spleen, lung, and kidney) were harvested for H&E staining. Moreover, the blood of the mice was collected for blood routine examination, including the RBCs, WBCs, HGB, PLT, and blood biochemistry examination, including ALT, AST, ALP, and CREA. Mice were monitored daily for signs of distress, including but not limited to: severe weight loss (>20%), lethargy, impaired mobility, hunched posture, ruffled fur, and neurological abnormalities (e.g., paralysis, circling behavior, ataxia). Throughout the study period, no neurological symptoms were observed in any treatment group. The predefined humane endpoint for euthanasia was a tumor volume exceeding 1500 mm^3^, body weight loss exceeding 20% of initial body weight, or the manifestation of any severe clinical signs listed above (including neurological symptoms).

### 4.14 Flow Cytometry

Zombie Dye is a fixable viability marker that irreversibly binds primary amines on cellular proteins, allowing live cells with intact membranes to exclude the dye (producing dim fluorescence) while dead cells with compromised membranes are intensely stained due to dye penetration and intracellular binding. Crucially, this fluorescent signal is retained after fixation and permeabilization, making it essential for our multi‐step flow cytometry analysis of tumor‐infiltrating immune cells. Briefly, tumors were digested to single‐cell suspensions and incubated with Zombie NIR dye (1:1000 in PBS) for 15 min at room temperature, a step that must be performed first to accurately discriminate live cells before any surface staining. Following two washes, cells were blocked and stained for surface markers, including CD80^+^and CD86^+^ to identify mature dendritic cells, as well as CD4 and CD8 to delineate T cell subsets. After 30 min incubation at 4°C, cells were fixed with 4% PFA, permeabilized with 0.1% saponin, and intracellularly stained for IFN‐γ for 60 min at 4°C to assess cytokine production. Our gating strategy sequentially selected live (Zombie‐negative) cells, then DCs (CD80^+^ and CD86^+^), followed by T cells (CD4^+^ and CD8^+^) to quantify IFN‐γ^+^ populations, ensuring that our analysis of DC activation and T cell function reflects only viable, functional immune cells.

### 4.15 ELISA

Blood samples of mice were collected from the orbital venous plexus into non‐anticoagulant tubes. Serum was separated by centrifugation at 3000 × g for 10 min at 4°C, then aliquoted and stored at ‐80°C to avoid repeated freeze‐thaw cycles. The concentrations of IL‐6 and TNF‐α in serum were determined using commercial ELISA kits. Briefly, all reagents and serum samples were equilibrated to room temperature for 30 min. A total of 100 µL of standard solutions or diluted serum samples (1:2 dilution with sample diluent) were added to the pre‐coated microplate wells, followed by incubation at 37°C for 1 h. After discarding the well contents, the plate was washed 3–4 times with washing buffer. Next, 100 µL of biotinylated detection antibody was added to each well and incubated at 37°C for 30 min. After another round of washing, 100 µL of streptavidin‐horseradish peroxidase conjugate was added, and the plate was incubated at 37°C for 15 min. Following the final washing step, 100 µL of TMB substrate solution was added to each well, and the plate was incubated in the dark at room temperature for 10 min. The reaction was terminated by adding 50 µL of stop solution, and the absorbance at 450 nm was measured using a microplate reader. The concentrations of IL‐6 and TNF‐α in samples were calculated based on the standard curve generated from the standard solutions.

### 4.16 Statistical Analysis

Statistical analysis was performed using GraphPad Prism 9.0. All data were assessed for normality and homogeneity of variance prior to analysis. Continuous variables are presented as means ± SD. Sample sizes (n) are indicated in the figure legends, representing at least three independent experiments. For comparisons between two groups, a two‐sided Student's t‐test was used. For comparisons among multiple groups, one‐way analysis of variance (ANOVA) was performed, followed by Tukey's post‐hoc test for multiple comparisons. The significance level (α) was set at 0.05. A *p*‐value < 0.05 was considered statistically significant. Significance levels are denoted in the figures as ns (not significant, *P* ≥ 0.05), ^*^ *p* < 0.05, ^**^ *p* < 0.01, and ^***^ *p* < 0.001.

## Funding

This study was funded by the Natural Science Foundation of Hunan Province for Young Researchers (Grant No. 2025JJ60589), the Key Project of Hunan Provincial Department of Education (Grant No. 24A0289), the Climb Plan of Hunan Cancer Hospital (Grant No. ZX2021005), the Hunan Provincial Natural Science Foundation of China (Grant No. 2025JJ80824), the China Primary Health Care Foundation (Grant No. cphcf‐2023‐056), the Beijing Science and Technology Innovation Medical Development Foundation (Grant No. KC2023‐JX‐0082‐05), Hunan Cancer Hospital's Qihang Youth Fund (Grant No. QH2023011), and the Hunan Provincial Key Laboratory of Basic and Clinical Pharmacological Research on Gastrointestinal Tumors (Grant No. 2023TP1014).

## Conflicts of Interest

The authors declare no conflicts of interest.

## Ethics Approval Statement

This study was approved by the Institutional Animal Care and Use Committee (IACUC) of the University of South China (Ethics approval number: 0159‐2).

## Patient Consent Statement

This study involves no patient participation and does not use any human specimens, thus no patient consent is required.

## Supporting information




**Supporting File**: advs75225‐sup‐0001‐SuppMat.docx.

## Data Availability

The data that support the findings of this study are available from the corresponding author upon reasonable request.
