# Supplementary material for: Dual‐Physical‐Field Nanocatalysis: Injectable Hydrogel Enables Piezo‐Photothermal Synergy for Breast Cancer Therapy
Source: Adv Sci (Weinh). 2026 Apr 13:e22447. Online ahead of print. doi: 10.1002/advs.202522447 (PMC13334646; doi:10.1002/advs.202522447)
Supplement: Supplementary file 1 — Supporting File: advs75225‐sup‐0001‐SuppMat.docx. [file ADVS-9999-e22447-s001.docx]

**Supporting Information**

**Lap shear test.** The tissue adhesion strength of the hydrogel was evaluated using a lap shear adhesion test on freshly excised porcine skin. Briefly, porcine skin was cut into appropriate sizes. The BiOCl@CuO/F127@rGO hydrogel was applied between two pieces of porcine skin with an adhesion area of 10 mm × 10 mm and a thickness of 1 mm. After gelation at 37 °C, the samples were subjected to a tensile test using a universal testing machine (MTS C41) at a speed of 5 mm/min until failure at the adhesion interface. The adhesion strength was calculated as the maximum load divided by the bonding area. Three independent measurements were performed for each sample.

Table S1. Comprehensive Physicochemical and Mechanical Properties of BiOCl@CuO/F127@rGO Hydrogel.

| **Parameter** | **Value (mean ± SD)** | **Measurement Method** |
| --- | --- | --- |
| Electrical Conductivity | 4,250 ± 180 S·m⁻¹ (with rGO) 3,500 ± 150 S·m⁻¹ (without rGO) | Four-point probe at 25°C |
| Gelation Temperature | 23.5 ± 0.5°C (G' = G'' crossover) | Oscillatory rheometry (γ = 1%, f = 1 Hz) |
| Storage Modulus (G') | 850 ± 50 Pa (at 37°C, gel state) | Rheometry at 1 Hz |
| Loss Modulus (G'') | 180 ± 20 Pa (at 37°C, gel state) | Rheometry at 1 Hz |
| Complex Viscosity | 0.8 ± 0.1 Pa·s (at 37°C) 0.15 ± 0.05 Pa·s (shear-thinning at 100 s⁻¹) | Steady shear sweep |
| Injectability | Passes through 26 G needle at 25°C | Manual injection test |
| Adhesive strength | 12.5 ± 2.3 kPa | Lap shear test |


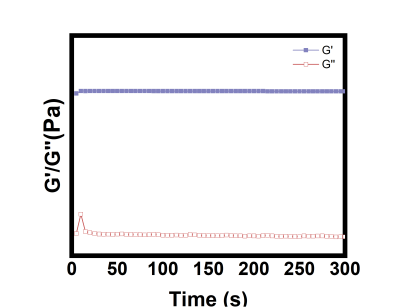

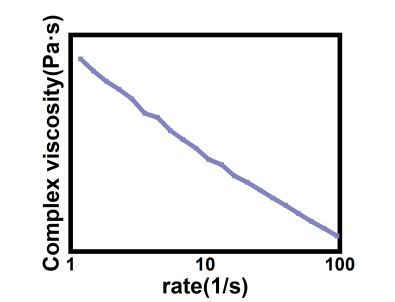

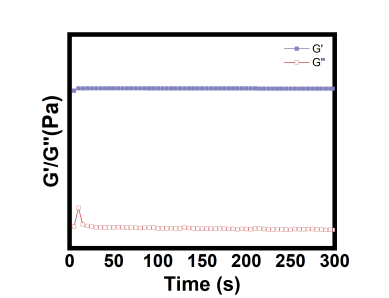


C

B

A

Figure S1. Supplementary rheological characterization of BiOCl@CuO/F127@rGO hydrogel. (A) Time-dependent oscillatory sweep at a fixed strain (1%) and frequency Hz, showing the mechanical stability of the viscoelastic network.(B) Steady shear sweep illustrating the complex viscosity as a function of shear rate, demonstrating prominent shear-thinning behavior.(C) Frequency sweep from 0.1 to 10 Hz at 37°C, where the dominance of G' over G'' indicates a robust, elastic-dominated gel state.


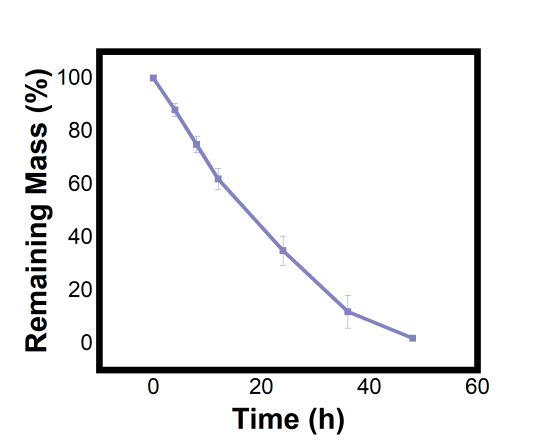

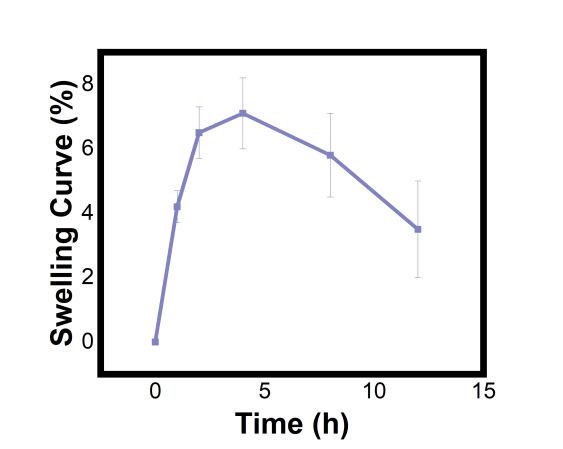


A

B

Figure S2. In vitro degradation and swelling characterization. (A) Degradation curve of the BiOCl@CuO/F127@rGO hydrogel in PBS (pH 7.4) at 37°C over 48 hours, showing the percentage of remaining mass over time (n = 3).(B) Swelling ratio curve of the hydrogel over 12 hours (n = 3).


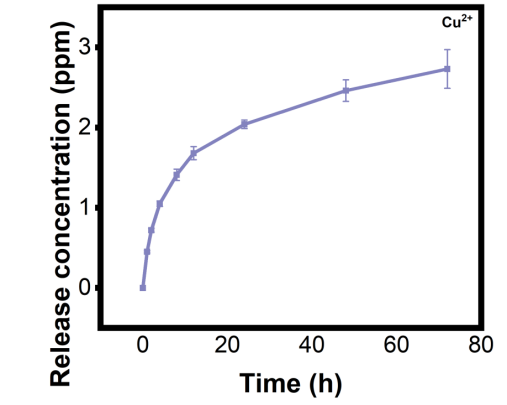


Figure S3. Quantitative evaluation of ion release. Cumulative release concentration of Cu^2+^ from the BiOCl@CuO/F127@rGO hydrogel over 72 hours measured by ICP-MS in PBS (pH 7.4) at 37 °C (n = 3).


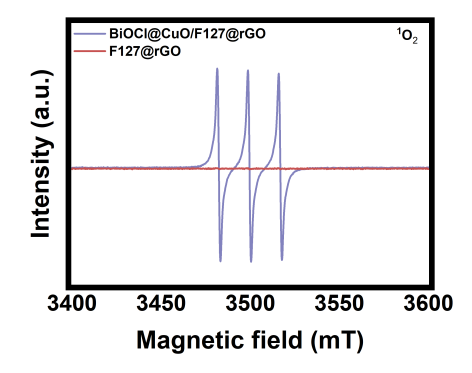


Figure S4.ESR spectroscopy using TEMP as a trapping agent under ultrasound (US) irradiation of F127@rGO and BiOCl@CuO@F127@rGO.


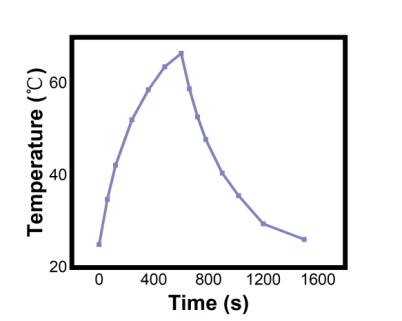

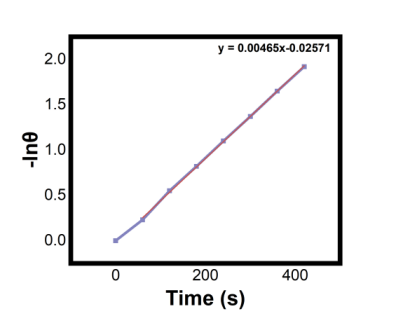

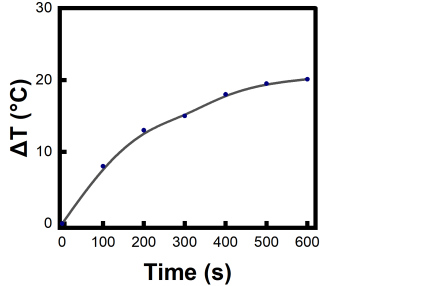


B

C

A

Figure S5. Photothermal performance and conversion efficiency of the hydrogels. (A) Photothermal heating and cooling profiles of the BiOCl@CuO/F127@rGO hydrogel under 808 nm NIR laser irradiation (1.0 W/cm^2^) for 600 s, followed by natural cooling to ambient temperature; (B) Linear regression of cooling time versus -\lnθ obtained from the cooling stage in (A); (C) Temperature of different time of F127@rGO under 808 nm NIR laser irradiation (1.0 W/cm^2^) for 600 s.


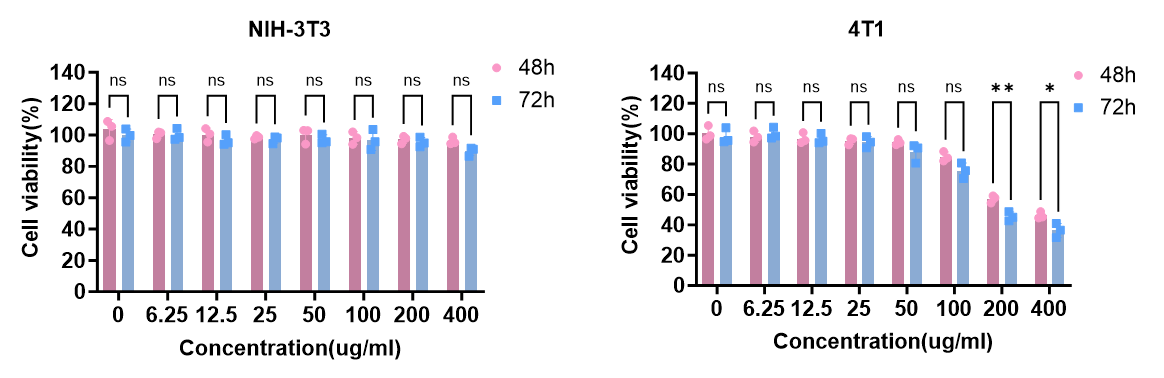


Figure S6. Cell viability of NIH-3T3 and 4T1 cells after treatment with BiOCl@CuO/F127@rGO at 48 h and 72h (n = 3). The data are presented as the means ± SD. The p-values were calculated via one-way analysis of variance (ANOVA), ns>0.05, *p < 0.05, **p < 0.01.

Table S2. Tumor remission rate in different groups.

|  | Control | F127@rGO hydrogel+US+NIR | BiOCl@CuO/F127@rGO hydrogel | BiOCl@CuO/F127@rGO hydrogel+US+NIR |
| --- | --- | --- | --- | --- |
| Tumor remission rate (%) | 0 | 8.4 | 58 | 90 |

Tumor remission rate (%) = [1-tumor weight (treated)/tumor weight (control)] × 100 %.

Figure S7. The release of high mobility group protein 1 (HMGB1) within the tumor (n = 3). The data are presented as the means ± SD. The p-values were calculated via one-way analysis of variance (ANOVA), ns>0.05 and ****p < 0.0001.

Figure S8. Body weight of mice in different groups after treatments over the 14-day study period (n = 5).

Figure S9. Blood biochemical indexes of different administration groups (n = 3).

Figure S10. Blood routine of different administration groups (n = 3).
